# Supplementary material for: Alternative Evolutionary Pathways in Paspalum Involving Allotetraploidy, Sexuality, and Varied Mating Systems
Source: Genes (Basel). 2023 May 24;14(6):1137. doi: 10.3390/genes14061137 (PMC10298031; doi:10.3390/genes14061137)
Supplement: Supplementary file 1 [file genes-14-01137-s001.zip › genes-2409653-supplementary.pdf]

Table S1. Number and percentage of ovules observed bearing different embryo sacs types in individual plants of five populations of four *Paspalum* species.

Species	Population	Plant	Ovules (no.)	Number (%) of ovules with			
				MES	AES	MES + AES	AbES
P. durifolium	PD1	1	30	27(90.0)	-	-	3(10.0)
		2	30	22(73.3)	-	-	8(26.7)
		3	31	28(90.3)	-	1(3.2)	2(6.5)
		4	30	21(70.0)	-	-	9(30.0)
		5	31	29(93.5)	-	-	2(6.5)
	PD2	1	30	28(93.3)	-	-	2(6.7)
		2	30	27(90.0)	-	-	3(10.0)
		3	30	29(96.7)	-	-	1(3.3)
		4	30	29(96.7)	-	-	1(3.3)
		5	30	28(93.3)	-	-	2(6.7)
	PD3	1	30	28(93.3)	-	-	2(6.7)
		2	30	29(96.7)	-	-	1(3.3)
		3	31	25(80.6)	-	6(19.4)	-
		4	30	24(80.0)	-	-	6(20.0)
		5	30	28(93.3)	-	-	2(6.7)
	PD4	1	30	24(80.0)	-	6(20.0)	-
		2	30	15(50.0)	-	4(13.3)	11(36.7)
		3	33	30(90.9)	-	-	3(9.1)
		4	30	30(100)	-	-	-
		5	30	30(100)	-	-	-
	PD5	1	30	18(60.0)	-	3(10.0)	9(30.0)
		2	34	30(88.2)	-	1(2.9)	3(8.8)
		3	30	21(70.0)	-	-	9(30.0)
		4	30	24(80.0)	-	-	6(20.0)
		5	32	31(96.9)	-	1(3.1)	-
P. ionanthum	PI1	1	31	28(90.3)	-	-	3(9.7)
		2	30	26(86.7)	-	3(10.0)	1(3.3)
		3	34	33(97.19	-	1(2.9)	-
		4	30	30(100)	-	-	-
		5	33	33(100)	-	-	-
	PI2	1	30	27(90.0)	-	2(6.7)	1(3.3)
		2	30	30(100)	-	-	-
		3	31	30(96.8)	-	-	1(3.2)
		4	30	30(100)	-	-	-
		5	30	30(100)	-	-	-
	PI3	1	30	29(96.7)	-	-	1(3.3)
		2	30	30(100)	-	-	-
		3	30	30(100)	-	-	-
		4	30	30(100)	-	-	-
		5	30	30(100)	-	-	-
	PI4	1	31	30(96.8)	-	-	1(3.2)
		2	37	36(97.3)	-	-	1(2.7)
		3	30	29(96.7)	-	-	1(3.3)
		4	35	34(97.1)	-	-	1(2.9)

		5	32	31(96.9)	-	-	1(3.1)
	PI5	1	36	35(97.2)	-	-	1(2.8)
		2	30	30(100)	-	-	-
		3	32	31(96.9)	-	-	1(3.1)
		4	30	30(100)	-	-	-
		5	30	30(100)	-	-	-
P. regnellii	PR1	1	30	20(66.7)	-	-	10(33.3)
		2	31	23(74.2)	-	-	8(25.8)
		3	30	27(90.0)	-	-	3(10.0)
		4	39	18(46.2)	-	-	21(53.8)
		5	30	24(80.0)	-	-	6(20.0)
	PR2	1	30	28(93.3)	-	-	2(6.7)
		2	31	19(61.3)	-	-	12(38.7)
		3	30	28(93.3)	-	-	2(6.7)
		4	30	28(93.3)	-	-	2(6.7)
		5	30	29(96.7)	-	-	1(3.3)
	PR3	1	30	26(86.7)	-	-	4(13.3)
		2	30	29(96.7)	-	-	1(3.3)
		3	32	31(96.9)	-	-	1(3.1)
		4	32	31(96.9)	-	-	1(3.1)
		5	31	31(100)	-	-	-
	PR4	1	34	33(97.1)	-	-	1(2.9)
		2	37	35(94.6)	-	-	2(5.4)
		3	34	32(94.1)	-	-	2(5.9)
		4	33	30(90.9)	-	-	3(9.1)
		5	32	32(100)	-	-	-
	PR5	1	37	36(97.3)	-	-	1(2.7)
		2	30	30(100)	-	-	-
		3	34	30(88.2)	-	-	4(11.8)
		4	33	30(90.9)	-	-	3(9.1)
		5	30	27(90.0)	-	-	3(10.0)
P. urvillei	PU1	1	34	32(94.1)	-	-	2(5.9)
		2	40	39(97.5)	-	-	1(2.5)
		3	30	29(96.7)	-	-	1(3.3)
		4	33	33(100)	-	-	-
		5	30	27(90.0)	-	-	3(10.0)
	PU2	1	32	29(90.6)	-	-	3(9.4)
		2	35	35(100)	-	-	-
		3	30	30(100)	-	-	-
		4	30	29(96.7)	-	-	1(3.3)
		5	31	30(96.8)	-	-	1(3.2)
	PU3	1	30	30(100)	-	-	-
		2	30	30(100)	-	-	-
		3	30	30(100)	-	-	-
		4	33	32(97.0)	-	-	1(3.0)
		5	33	33(100)	-	-	-
	PU4	1	31	30(96.8)	-	-	1(3.2)
		2	33	33(100)	-	-	-
		3	30	30(100)	-	-	-

	4	32	30(93.8)	-	-	2(6.2)
	5	30	28(93.3)	-	-	2(6.7)
PU5	1	30	30(100)	-	-	-
	2	30	30(100)	-	-	-
	3	35	30(100)	-	-	-
	4	30	30(100)	-	-	-
	5	30	30(100)	-	-	-

MES: meiotic embryo sac, AES: aposporous embryo sac; MES+AES: ovules bearing meiotic and aposporous embryo sacs, AbES: absent or undeveloped embryo sac.

.

Table S2. Number of pollinated spikelets and seed set for two pollination conditions (self- and open-pollination) during two flowering periods (1st, 2015-2016 and 2nd, 2016-2017) in populations of four Paspalum species.

**Species**	**Population**	**Pollination method**	**Period**	**Pollinated Spikelets (no.)**	**Seed set (%)**			
					**Mean**	**Min.**	**Max.**	**CV**
P. durifolium	PD1	Self-pollination	1st	13,870	0.27	0.00	0.49	74.63
			2nd	12,447	0.61	0.00	1.46	102.00
		Open-pollination	1st	18,155	28.09	13.84	38.50	34.11
			2nd	19,620	29.58	10.10	44.10	43.04
	PD2	Self-pollination	1st	12,510	0.35	0.00	1.15	147.77
			2nd	17,633	0.65	0.20	1.10	62.46
		Open-pollination	1st	17,537	46.30	39.80	60.30	17.88
			2nd	23,034	51.54	43.30	60.00	14.66
	PD3	Self-pollination	1st	20,534	0.16	0.00	0.58	145.17
			2nd	17,967	0.65	0.04	2.10	133.32
		Open-pollination	1st	18,470	57.90	45.87	75.91	20.12
			2nd	24,338	45.16	19.00	69.40	42.75
	PD4	Self-pollination	1st	9,538	1.24	0.05	4.45	148.39
			2nd	10,729	0.47	0.00	1.50	128.02
		Open-pollination	1st	13,059	45.52	6.14	95.37	83.83
			2nd	14,763	42.70	30.00	64.00	34.75
	PD5	Self-pollination	1st	10,632	0.17	0.00	0.48	129.51
			2nd	20,406	1.18	0.06	3.20	107.46
		Open-pollination	1st	20,748	33.17	17.6	51.22	42.53
			2nd	23,408	27.97	18.13	42.60	32.28
P. ionanthum	PI1	Self-pollination	1st	896,00	1.00	0.00	3.60	152.88
			2nd	600,00	1.00	0.00	3.60	152.26
		Open-pollination	1st	1,402	35.60	20.00	49.60	31.79
			2nd	1,974	23.20	3.50	39.20	61.74
	PI2	Self-pollination	1st	1,146	0.26	0.00	1.33	223.61

			2nd	2,289	1.22	0.00	3.58	139.84
		Open-pollination	1st	2,186	60.25	44.92	80.71	22.59
			2nd	4,288	31.27	15.33	51.27	44.79
	PI3	Self-pollination	1st	1,315	4.26	0.00	21.27	223.61
			2nd	1,324	1.36	0.00	3.55	118.00
		Open-pollination	1st	2,533	49.66	42.00	58.36	13.06
			2nd	1,143	58.88	29.22	75.47	29.89
	PI4	Self-pollination	1st	1,214	0.16	0.00	0.58	223.61
			2nd	2,549	1.10	0.00	2.91	109.53
		Open-pollination	1st	1,608	52.80	19.76	68.46	38.24
			2nd	2,970	39.22	30.63	46.70	15.39
	PI5	Self-pollination	1st	1,261	2.85	0.00	9.02	123.99
			2nd	1,969	0.71	0.00	2.21	122.51
		Open-pollination	1st	1,711	45.53	11.29	64.40	47.33
			2nd	2,444	27.82	15.58	41.88	42.23
P. regnellii	PR1	Self-pollination	1st	15,139	8.51	0.96	15.35	73.56
			2nd	18,527	4.96	0.33	18.13	151.33
		Open-pollination	1st	14,894	23.47	9.22	49.76	72.02
			2nd	18,481	34.19	16.61	58.51	52.63
	PR2	Self-pollination	1st	13,811	25.93	12.00	46.31	54.58
			2nd	17,928	6.19	2.62	12.95	69.72
		Open-pollination	1st	16,890	38.14	17.68	62.96	43.30
			2nd	15,929	44.17	18.33	68.70	41.93
	PR3	Self-pollination	1st	12,753	12.44	0.45	31.24	101.83
			2nd	11,263	9.74	3.62	24.75	89.48
		Open-pollination	1st	20,919	24.06	12.40	41.37	52.51
			2nd	18,050	28.70	9.73	62.12	72.44
	PR4	Self-pollination	1st	12,584	10.48	1.17	29.58	110.58
			2nd	15,553	2.34	0.16	4.83	82.49
		Open-pollination	1st	15,157	35.83	11.01	54.41	44.93
			2nd	18,991	20.77	1.52	35.48	63.21
	PR5	Self-pollination	1st	12,280	25.16	11.58	37.30	41.70
			2nd	12,978	12.47	0.13	18.34	56.92
		Open-pollination	1st	15,833	62.20	44.41	85.16	25.00
			2nd	15,765	37.35	0.36	63.31	69.19
P. urvillei	PU1	Self-pollination	1st	9,993	44.61	20.56	84.17	56.86
			2nd	14,976	40.75	12.06	59.80	43.60
		Open-pollination	1st	20,346	80.15	72.64	88.53	8.12
			2nd	29,845	59.76	31.86	83.20	30.82
	PU2	Self-pollination	1st	9,029	30.76	1.47	58.60	67.84
			2nd	14,994	52.99	36.37	63.98	22.00
		Open-pollination	1st	14,871	86.87	72.22	92.83	9.78
			2nd	31,962	74.41	44.00	93.80	29.39
	PU3	Self-pollination	1st	9,233	38.47	11.10	71.88	68.40
			2nd	13,788	32.75	20.90	50.48	37.07
		Open-pollination	1st	8,841	92.01	84.77	96.48	5.53
			2nd	21,562	64.01	51.47	89.25	25.76
	PU4	Self-pollination	1st	10,436	33.34	6.88	75.80	75.79
			2nd	14,567	52.22	41.10	68.40	20.77

	Open-pollination	1st	13,736	76.71	55.83	95.19	21.07
		2nd	29,181	69.56	64.85	75.09	6.02
PU5	Self-pollination	1st	10,020	31.80	21.70	40.20	24.74
		2nd	11,706	54.44	26.05	71.70	39.28
	Open-pollination	1st	11,910	74.00	56.90	91.70	18.34
		2nd	23,105	66.05	52.00	85.70	21.41
